# The Prevalence of Behavioural Symptoms and Psychiatric Disorders in Hadza Children

**DOI:** 10.1038/s41598-023-48114-4

**Published:** 2023-12-12

**Authors:** Dennis Ougrin, Emma Woodhouse, Gavin Tucker, Amy Ronaldson, Ioannis Bakolis

**Affiliations:** 1 Youth Resilience Unit, Centre for Psychiatry and Mental Health, Wolfson Institute of Population Health, WHO Collaborating Centre for Mental Health Services Development, Queen Mary University of London, London, UK; 2 Compass Psychology Services, Bromley, Kent, UK; 3 Department of Forensic and Neurodevelopmental Sciences, Institute of Psychiatry, Psychology and Neuroscience, King’s College London, London, UK; 4 South London and Maudsley NHS Foundation Trust, London, UK; 5 Health Service and Population Research Department, Institute of Psychiatry, Psychology and Neuroscience, King’s College London, London, UK; 6 Department of Biostatistics and Health Informatics, Institute of Psychiatry, Psychology and Neuroscience, King’s College London, London, UK

**Keywords:** Addiction, ADHD, Anxiety, Autism spectrum disorders, Bipolar disorder, Depression, Obsessive compulsive disorder, Post-traumatic stress disorder, Psychosis, Schizophrenia

## Abstract

The worldwide pooled prevalence of psychiatric disorders in children is 13.4%. Studying the prevalence of childhood psychiatric disorders across radically different economic systems and social structures could indicate universal factors leading to their development. The prevalence of childhood psychiatric disorders in a mixed-subsistence foraging society has not been studied. The Strengths and Difficulties Questionnaire and the Development and Well-Being Assessment were used to compare the prevalence of behavioural symptoms and psychiatric disorders in Hadza children aged 5–16 years (n = 113) to a nationally representative sample from England (n = 18,029) using a cross-sectional study design. Emotional problems, conduct problems and hyperactivity were lower in the Hadza children. Prosocial behaviour and peer problems were higher in Hadza children. 3.6% of Hadza children met the criteria for a psychiatric disorder compared to 11.8% of English children. All psychiatric disorders in Hadza children were co-morbid with autism spectrum disorder. No child from the Hadza group met the criteria for an emotional, behaviour or eating disorder. Further work should study the factors which lead to the different prevalence of psychiatric disorders in Hadza children.

Psychiatric disorders in children and adolescents are common, treatable, and growing in prevalence in England^1^. The worldwide pooled prevalence of psychiatric disorders in children and adolescents is 13.4%^2^. The worldwide pooled prevalence of anxiety disorders, depressive disorders, attention-deficit hyperactivity disorder (ADHD) and disruptive disorders are 6.5%, 2.6%, 3.4% and 5.7% respectively^2^. The global prevalence of autism spectrum disorder (ASD) is 1%^3^. 73.9% of all psychiatric disorders start before the age of 18^4^, with the peak onset at 14–15 years. The onset of psychiatric disorders involves complex interactions between genetic^5^, cognitive, environmental and societal factors^6–8^ but there is little agreement about the necessary or sufficient conditions to cause psychiatric disorders. Previous work has shown that sample representativeness, sampling frame and diagnostic method are significant moderators of prevalence estimates of childhood psychiatric disorders however this does not completely account for the observed variance^2^. Best practice in case definition involves the presence of functional impairment in addition to symptoms^9^, and the ability of social structures to make accommodations for psychiatric symptoms can reduce functional impairment and thus lower the rates of diagnosable psychiatric disorders. Studies to date have taken place in agricultural societies but the prevalence of childhood psychiatric disorders in the radically different economic system and social structure of a mixed-subsistence foraging tribe are entirely unknown^10^. Studying the prevalence of psychiatric disorders in a mixed-subsistence foraging tribe could provide some indication as to universal mechanisms underlying childhood psychiatric disorders.

The Hadza are an indigenous ethnic group of mixed-subsistence foragers residing in Northern Tanzania in a savannah woodland habitat south of the Serengeti. The total population of the Hadza is approximately 1000–1500 individuals, with approximately 300–400 children. Calculating the exact number of Hadza children is difficult, as no full census has been carried out, and records of birth dates are sparse. Historically, the Hadza have largely subsisted by practicing a hunter-gatherer lifestyle. It is impossible to tell how representative this group is of ancestral cultural and social systems. However, they operate an economic system and social organisation which is radically different to those seen in agricultural societies, and is relatively closer to living conditions in the Paleolithic era than contemporary Western populations^11^.

All members of the Hadza ethnic group speak Hadzane as their first language and almost all adults speak Swahili as their second language. While the vast majority of Hadza individuals are non-literate and have not attended school, most people above the age of 8 years old speak Swahili. Individuals residing in bush camps (located at least one day’s walk from a village) consume a diet that is predominantly composed of wild foods but increasingly includes domesticated cultigens such as maize—largely consumed as supplemental foods in the diet. As is true elsewhere in Northern Tanzania, Hadza women experience high levels of infant and maternal mortality, with lack of access to professional health care services, including reproductive health services^12,13^.

There have been previous studies attempting to understand aspects of mental health and wellbeing of the Hadza. The mental health of postpartum females of Hadza mixed-subsistence foragers has been studied in one previous study^14^. In addition, the Hadza perception of emotions has been studied^15^. There is a large body of work in medical anthropology detailing problems with applying universal diagnostic methodologies and treatments wholesale across populations^16^. However, no studies have compared the prevalence of childhood behavioural symptoms and psychiatric disorders in mixed-subsistence forager societies versus Western ones. We aimed to investigate the prevalence of paediatric behavioural symptoms and psychiatric disorders in the Hadza population, compared to a nationally representative sample from England, an agricultural society. We hypothesised that the wide differences in important economic, social and cultural factors in Hadza society compared to agricultural societies could lead to differences in the prevalence of childhood psychiatric disorders.

## Results

### Mental health of Hadza children and MHCYP comparator groups

**Figure 1:**
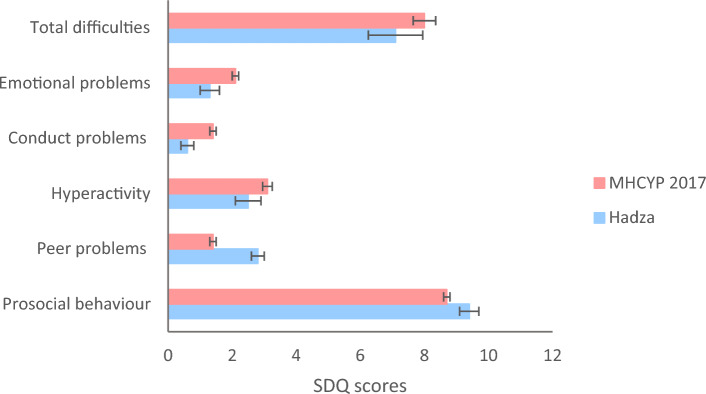
Comparison between the Hadza tribe and from the Mental Health of Children and Young People in England 2017 (MHCYP 2017) survey on total SDQ scores and SDQ subscales for ages 5–16.

SDQ scores for the overall sample (n = 140), the subsample aged 5–16 years (n = 113), and for 5–16-year-olds from MHCYP 2017 (n = 2588) are presented in Table 1. As inferential statistics were not possible due to the summary nature of the MHCYP data, descriptive comparisons were made between 5 and 16-year-olds from the Hadza tribe and 5–16-year-olds from the MHCYP which are presented in Fig. 1.

**Table 1:** Means and 95% confidence intervals of Hadza children and of MHCYP 2017 survey.

	Overall sample (N = 140)	Aged 5–16y (N = 113)	MHCYP 2017 Aged 5–16y (N = 2588)
	*M, 95% CI*	*M, 95% CI*	*M, 95% CI*
Total difficulties	6.84, 6.12–7.57	7.14, 6.29–8.00	8.0, 7.7–8.4
Emotional problems	1.20, 0.93–1.48	1.26, 0.94–1.57	2.1, 2.0–2.2
Conduct problems	0.49, 0.32–0.66	0.57, 0.37–0.78	1.4, 1.3–1.5
Hyperactivity	2.39, 2.04–2.75	2.52, 2.11–2.93	3.1, 3.0–3.3
Peer problems	2.77, 2.59–2.94	2.79, 2.58–2.99	1.4, 1.3–1.5
Prosocial	9.43, 9.20–9.67	9.40, 9.13–9.68	8.7, 8.6–8.8
Boys*	(N = 50)	(N = 39)	(N = 1239)
Total difficulties	5.71, 4.84–6.58	5.81, 4.80–6.81	8.6, 8.1–9.1
Emotional problems	0.88, 0.49–1.27	0.85, 0.41–1.28	1.9, 1.8–2.1
Conduct problems	0.18, 0.06–0.30	0.21, 0.05–0.36	1.5, 1.4–1.7
Hyperactivity	1.70, 1.18–2.22	1.72, 1.10–2.33	3.6, 3.4–3.8
Peer problems	2.95, 2.70–3.19	3.04, 2.76–3.32	1.5, 1.4–1.7
Prosocial	9.94, 9.85–10.03	9.92, 9.81–10.04	8.4, 8.3–8.6
Girls*	(N = 57)	(N = 44)	(N = 1349)
Total difficulties	6.22, 4.91–7.53	6.54, 4.88–8.20	7.4, 7.1–7.8
Emotional problems	1.12, 0.59–1.65	1.34, 0.68–2.01	2.3, 2.1–2.4
Conduct problems	0.35, 0.05–0.66	0.44, 0.04–0.83	1.2, 1.1–1.3
Hyperactivity	1.89, 1.38–2.41	1.94, 1.30–2.57	2.7, 2.5–2.8
Peer problems	2.85, 2.56–3.13	2.83, 2.50–3.15	1.3, 1.2–1.4
Prosocial	9.28, 8.78–9.78	9.27, 8.64–9.90	9.0, 8.9–9.1

*Information about sex was gathered for 107 CYP from the overall sample and 83 from the subsample aged 5–16 years. SDQ data for this subsample are presented here.

Children from the Hadza tribe (M = 7.1, 95% CI 6.3–8.0) had lower levels of total difficulties compared to children from MHCYP (M = 8.0, 95% CI 7.7–8.4). However, as the confidence intervals overlap it is unlikely that this difference was statistically significant. Levels of emotional problems (Hadza: M = 1.3, 95% CI 0.9–1.6; MHCYP 2017: M = 2.1, 95% CI 2.0–2.2), conduct problems (Hadza: M = 0.6, 95% CI 0.4–0.8; MHCYP : M = 1.4, 95% CI 1.3–1.5) and hyperactivity (Hadza: M = 2.5, 95% CI 2.1–2.9; MHCYP 2017: M = 3.1, 95% CI 3.0–3.3) were lower in the Hadza children, and levels of prosocial behaviour (Hadza: M = 9.4, 95% CI 9.1–9.7; MHCYP 2017: M = 8.7, 95% CI 8.6–8.8) were higher, compared to children in MHCYP 2017. However, peer problems appeared to be higher among Hadza children (Hadza: M = 2.8, 95% CI 2.6–3.0; MHCYP 2017: M = 1.4, 95% CI 1.3–1.5). On all SDQ subscales, confidence intervals did not overlap between Hadza children and children from the MHCYP indicating that these differences are likely to be statistically significant.

### Gender differences in positive and negative behavioural symptoms (SDQ scores)

Looking at boys and girls separately (Table 1) revealed that differences in SDQ scores between Hadza children and English children were more pronounced in boys. Boys from the Hadza tribes (compared to boys from MHCYP 2017) appeared to have significantly (confidence intervals do not overlap) lower total difficulty scores, lower levels of emotional and conduct problems, lower levels of hyperactivity, and higher levels of prosocial behaviour. However, peer problems were higher in boys from the Hadza tribe.

Overlaps in confidence intervals suggest that girls from the Hadza tribe did not differ significantly from girls in England in total difficulty scores, levels of hyperactivity, and levels of prosocial behaviour. However, they had lower levels of emotional and conduct problems, and higher levels of peer problems when compared with children in MHCYP 2017.

### Associations between sociodemographic factors and SDQ scores in Hadza children

Sociodemographic details are recorded in Table 2. We examined associations between age, sex, camp, and living status (living with father, living with mother) and SDQ scores in in the overall sample (aged 3–17 years, n = 107) from the Hadza tribe. There was no evidence that age was associated with total SDQ scores or any SDQ subscale (all *p* values > 0.05). Sex was not associated with any SDQ score apart from prosocial behaviour (β = − 0.66, 95% CI − 1.19 to − 0.13) where boys (*M* = 9.94, *SD* = 0.31) had significantly higher levels than girls (*M* = 9.28, *SD* = 1.88).

**Table 2:** Sample characteristics and results from the DAWBA assessment.

	Overall sample aged 3-17y (N = 107)	Subsample aged 5-16y (N = 83)
	*M* ± *SD, N(%)*	*M* ± *SD, N(%)*
Camp		
Camp 1	49 (45.8)	37 (44.6)
Camp 2	25 (23.4)	21 (25.3)
Camp 3	15 (14.0)	11 (13.2)
Camp 4	18 (16.8)	14 (16.9)
Age	8.50 ± 3.80 (n = 140) median = 8, IQR = 5–11	9.03 ± 3.31 (n = 113) median = 8, IQR = 6–11
Female	57 (53.3)	44 (53.0)
Lives with mother	75 (70.1)	56 (67.5)
Lives with father	64 (59.8)	47 (56.6)
Education	31 (29.0)	28 (33.7)
Years of education	4.14 ± 2.59 (n = 29)	4.19 ± 2.67 (n = 26)
Physical disability	3 (2.8)	2 (2.4)
Psychological problems	3 (2.8)	3 (3.6)
Alcohol consumption	0 (0.0)	0 (0.0)
Tobacco use	6 (5.6)	5 (6.0)
Marijuana use	4 (3.7)	3 (3.6)
Psychiatric diagnosis		
Autism spectrum disorder	4 (3.7)	3 (3.6)
ADHD	2 (1.9)	1 (1.2)
Social anxiety	1 (0.9)	1 (1.2)

*Note 1* We provide sociodemographic information for the overall sample (N = 107) aged 3–17 years. We also provide this information for the subsample of these children who were aged 5–16 years to facilitate comparison with the MHCYP.

*Note 2* The three psychiatric disorders listed in this table were the only psychiatric disorders detected in this sample, the prevalence of all other psychiatric disorders was zero. There was comorbidity of disorders, as explained in the “Results” section.

Summary SDQ scores for each Hadza camp are provided in Table 3 to facilitate comparison. Compared to children from Camp 1, children from Camp 2 had significantly lower total difficulty scores (β = − 3.17, 95% CI − 5.03 to − 1.31), whereas scores for the children from Camp 3 were significantly higher (β = 2.30, 95% CI 0.07–4.53). In terms of SDQ subscales, emotional problems were lower in Camp 2 (β = − 1.02, 95% CI − 1.80 to − 0.24) and higher in Camp 3 (β = 1.30, 95% CI 0.36–2.23) compared to children from Camp 1. Similarly, when compared with children from Camp 1, those from Camp 2 had lower levels of hyperactivity (β = − 1.71, 95% CI − 2.48 to − 0.94) and those from Camp 3 had higher levels (β = 1.73, 95% CI 0.80–2.65). Peer problems were lower in Camp 3 (β = − 0.71, 95% CI − 1.25 to − 0.17) and Camp 4 (β = − 0.80, 95% CI − 1.30 to − 0.29) compared to Camp 1. There were no significant differences in conduct problems or prosocial behaviour between camps.

**Table 3:** SDQ scores by Hadza camp (N = 107).

	Camp 1 (N = 49)	Camp 2 (N = 25)	Camp 3 (N = 15)	Camp 4 (N = 18)
	*M* ± *SD*	*M* ± *SD*	*M* ± *SD*	*M* ± *SD*
Total difficulties	6.69 ± 5.10	3.52 ± 1.23	8.99 ± 2.87	4.94 ± 2.34
Emotional problems	1.10 ± 2.09	0.08 ± 0.40	2.40 ± 1.35	0.89 ± 1.18
Conduct problems	0.43 ± 1.21	0.00 ± 0.00	0.41 ± 0.73	0.11 ± 0.32
Hyperactivity	2.03 ± 1.95	0.32 ± 0.56	3.76 ± 1.74	1.61 ± 1.24
Peer problems	3.13 ± 0.98	3.12 ± 1.05	2.42 ± 0.62	2.33 ± 0.77
Prosocial	9.27 ± 2.00	9.84 ± 0.55	9.73 ± 0.60	10.0 ± 0.00

There was no evidence that living with fathers compared to living with mothers was associated with total SDQ scores.

### Prevalence of mental disorders of Hadza and MHCYP comparator groups

From the Hadza dataset, DAWBA data were available for 61/80 children aged 5–10, and 22/33 children aged 11–16. 3.6% (n = 3) of children from the Hadza 5–16 year old subsample met the criteria for any psychiatric disorder, compared to 11.8% in the comparable age group from MHCYP. All Hadza children with psychiatric disorders in the 5–16 year sample were girls and all were from the 5–10 year old age group. All other psychiatric disorders diagnosed in Hadza children were comorbid with ASD. No children from the Hadza 11–16 age group met the criteria for a psychiatric disorder, compared to 14.4% in the comparable age group from MHCYP.

Regarding emotional disorders, no child from the Hadza met the criteria for an emotional disorder. In the MHCYP data, 4.1% of children aged 5–10, and 9.0% of children aged 11–16 met the criteria for a emotional disorder.

No child from the Hadza met the criteria for a behavioural disorder. In the MHCYP data, 5.0% of children aged 5–10, and 6.2% of children aged 11–16 met the criteria for a behavioural disorder.

No child from the Hadza met the criteria for an eating disorder. In the MHCYP data, 0.1% of children aged 5–10, and 0.6% of children aged 11–16 met the criteria for an eating disorder.

4.9% of Hadza children aged 5–10 met the criteria for ASD, compared to 1.5% of 5–10 year olds in MHCYP 2017. No Hadza children aged 11–16 met the criteria for ASD, compared to 1.2% of the same age group in MHCYP 2017.

The number of Hadza children with mental disorders was too small to be able to conduct meaningful statistical analysis, however here we provide a narrative description for illustrative purposes:

Child 1 was a female aged 6 and met the criteria for ASD. She displayed high emotional difficulties, close to average conduct problems, high hyperactivity problems, high peer problems, very low prosocial strengths, and her total difficulties score was very high.

Child 2 was a female aged 6 and met the criteria for ASD, ADHD, Rett’s syndrome, social phobia, and motor tics. She displayed very high emotional difficulties, high conduct problems, very high hyperactivity problems, close to average peer problems, very low prosocial strengths, and her total difficulties score was very high.

Child 3 was a female aged 10 and met the criteria for ASD, social phobia, and Tourette syndrome. She displayed very high emotional difficulties, very high conduct problems, slightly raised hyperactivity problems, high peer problems, very low prosocial strengths, and her total difficulties score was very high. We also noted she had characteristic clinical features of Down syndrome.

Child 4 was a male aged 4 and met the criteria for ASD and ADHD. He displayed close to average scores for emotional problems, conduct problems, hyperactivity, prosocial strengths and total difficulties. He displayed high peer problems scores.

## Discussion

This study examined the prevalence of behavioural symptoms and psychiatric disorders in the children of a mixed-subsistence foraging group. There has been no research into childhood psychiatric disorders in mixed-subsistence forager societies despite being a population of interest in investigating universal mechanisms of psychiatric disorders. We show that the overall prevalence of behavioural symptoms and psychiatric disorders in Hadza children appears lower that the prevalence of agricultural society such as England^17^, and lower than recent estimates of global prevalence^2^. However, the prevalence of ASD appears to be higher in Hadza children than the estimated global prevalence^3^.

Although we did not formally compare the prevalence of psychiatric disorders, we did not find evidence of any current disorder in male children (except for one boy aged 4 whom we excluded from the main analysis), and most domains of behavioural symptoms of psychiatric disorders were lower in Hadza boys compared to girls. It is difficult to make direct comparisons of strengths and difficulties between Hadza children and English children. This is because the SDQ is scored according to subjective accounts of strengths and functional impairments in a range of domains. It is possible that behaviours which would be considered impairments in a Western society could be viewed as neutral or valuable in Hadza society e.g. motor overactivity or fighting.

The prevalence of ASD in Hadza children and adolescents appears to be higher than in the West and the prevalence of behavioural and emotional disorders is lower. We found four young people with ASD. Based on clinical characteristics and symptoms, one child was likely to have Rett’s syndrome, one was likely to have Down syndrome and one child meeting the criteria for ASD did not have any obvious chromosomal abnormalities, albeit with a possible facial dysmorphia. Two out of three children with ASD also met the diagnostic criteria for ADHD. This increased prevalence of ASD is of interest especially given the low prevalence of other disorders. Cerebral malaria, consanguineous marriages, widespread alcohol misuse in adults including pregnant women, and many women giving birth relatively late in life might explain some of these findings.

We have diagnosed only a handful of children or adolescents with emotional disorders. Some participants reported a range of specific fears in their children. These include fears of animals, most commonly elephants and leopards, fears of supernatural creatures, most commonly ancestors’ spirits and witches, fears of certain groups of people, most commonly Datoogas (a nearby pastoralist population) and fears of aspects of natural environment, most commonly storms and thunder. There was no evidence of significant distress or dysfunction associated with these fears except in the children with ASD.

No diagnoses of current depressive disorders were made. There was one participant who reported a brief episode of depressive features in two of her children. Their symptoms included sadness, loss of energy, loss of interest, irritability, negative thoughts and poor sleep. The episode was associated with the children’s mother leaving the camp to search for food and a state of hunger.

We have found no evidence of self-harm. Older women are familiar with the idea of decorating their skin by burning it intentionally creating patterns. As well as considered beautiful, the patterns indicate strength and endurance. We did not observe these patterns in younger Hadza children. We observed self-injurious behaviour in the child with Rett’s syndrome and it was reported for the child with Down syndrome. The child with Rett’s syndrome hit her head with her hands repetitively when someone took away desirable objects from her.

We did not find evidence of eating disorders. Questions about eating disorders were met with smiles and incredulity. The Hadza spend considerable amount of time everyday hunting and gathering for food and the concept of a distorted body image appears entirely unfamiliar to them. The Hadza appeared unexposed to the idea of being thin as a desirable characteristic. Hadza men display a preference for women with a lower profile waist-to-hip ratio^18^. Many Hadza would consider an overweight man unlikely to be a good hunter or tree climber^19^.

Although we made no diagnoses of psychotic disorders, the concept of impaired reality-testing is familiar to the Hadza. During the study period we were aware of a man who was likely experiencing a psychotic episode, possibly induced by smoking cannabis. Community members reported that the man had been shooting at people with poisoned arrows. We were told he believed that they were trying to harm him, but there was no clear reason for this belief. This behaviour was described as bad “masalaka”.

This is the only study to date which has investigated behavioural symptoms and the prevalence of psychiatric disorders in children in a mixed-subsistence foraging tribe. Comprehensive screening tools with cross-cultural validity were used to investigate a large range of behaviours and mental disorders. The comparator group was a large nationally representative community sample of a modern society. As the Hadza population is small, it was possible to interview a large percentage of the overall population of Hadza children existing today. This means that the prevalence of behavioural symptoms and psychiatric disorders in our sample population is likely to resemble the prevalence in the overall population of Hadza children.

This study has several limitations. As there has been little research into the mental health of mixed-subsistence foraging populations, the ICD-10 system of diagnostic classification has not been validated in our sample population. However from narrative descriptions of tribe members’ understanding of mental illness, they appeared to describe discrete constructs of mental illness which broadly map onto some ICD-10 constructs of mental disorders. Due to marked economic, social and cultural variation across mixed-subsistence forager groups globally, it is difficult to draw conclusions about the prevalence of childhood psychiatric disorders in this system generally based on studying one group in isolation.

Hadza concepts of age do not neatly map onto Western constructs, and written records of birth dates are sparse. The concept of ageing is marked by features of physical development and thus it was difficult to accurately gauge the overall numbers of Hadza children. This also meant it was difficult to know the age of some Hadza children, which could be a confounding factor in the sample selection and results.

Sampling in the current study was limited by availability of participants as well as the time available for data collection. This opportunistic sampling technique may have introduced bias and led to a relatively small sample size. Moreover, the estimated population of Hadza children is much smaller than the sampling frame of MHCYP 2017. This makes direct comparisons of prevalence of mental disorders difficult as the Hadza prevalence rate is impacted far more by a small change in absolute numbers than in MHCYP 2017. Individual-level data for MHCYP 2017 was not available, which prevented direct comparisons of Hadza children with subgroups of interest in MHCYP 2017 e.g. children of very low socioeconomic status. Due to time and resource constraints, it was only possible to administer the parent/carer DAWBA. It was not possible to administer a DAWBA to all carers who completed an SDQ, this could impact the estimates of prevalence of psychiatric disorders.

The diagnosis of ASD is typically made using the Autism Diagnostic Observation Schedule (ADOS), or the Autism Diagnostic Interview-Revised (ADI-R). However, the DAWBA shows good specificity and sensitivity for diagnosing ASD in community samples^20^, and has been used in the MHCYP comparison sample^21^. Disorders such as ADHD are typically made on the basis of multiple informants, reporting on the child’s behaviours in environments such as home and school. However, as the Hadza children do not attend school, it was not possible to get informant data about behavioural problems in this setting.

The SDQ was administered verbally with the use of an interpreter, unlike the written SDQ in the MHCYP sample and this may impact the prevalence of behavioural symptoms in the Hadza group. However as the majority of Hadza are non-literate, it was not feasible to administer the written version. Additionally, the SDQ and DAWBA were administered with the use of an interpreter which could also impact the prevalence of psychiatric disorders in the Hadza group.

Our findings partially support the universality of mechanisms causing some childhood psychiatric disorders: while the prevalence of ASD is higher than in Westernised societies, we did not find evidence of childhood depressive disorders or behavioural disorders, and eating disorders are completely unheard of. We postulate that the reasons behind the low prevalence of most psychiatric disorders in this economic system and social organisation are likely to be complex. This could be due to several factors including the extraordinary community support we observed that the affected young people receive leading to lower functional impairment, more active lifestyles, differences in gut microbiome^22^, and differences in social cognition ^15^. Community support is one of the strongest known predictors of good mental health in young people^23^.

This is the first study investigating the prevalence of behavioural symptoms and mental disorders in the children of a mixed-subsistence foraging population. This is a cross-sectional study of prevalence of psychiatric symptoms and disorders, thus longitudinal study designs are warranted in order to acquire more accurate descriptions of mental health in Hadza children, and children in other mixed-subsistence forager groups globally. Further research is needed into the causes of disparities in the prevalence of mental disorders with established cohorts which could be observed over time in these population compared to children in Westernised societies.

## Methods

### Study design

Two authors (DO, a consultant child and adolescent psychiatrist, and EW, a neurodevelopmental specialist who is a trainer and supervisor in the ADOS and ADI-R) travelled to the region in northern Tanzania where Hadza people live during the dry season. One camp was identified as a starting position based on proximity to Arusha where the authors travelled from, and three further study sites were chosen according to opportunistic travel capabilities between camps. Given the low sample size and the risk of individual children being identifiable from descriptions, we have not named the four camps chosen for this study. No camp movements were observed during the study period. The sample size was determined by how many verbal administrations of established psychometric tests could be conducted in the available study time. On arrival to the camp, the researchers were introduced by the Swahili/Hadzane interpreter, and an explanation of the research was given to camp inhabitants. All children between the ages of approximately 3 and 17 years old residing in four Hadza camps were identified as the sample population. Each child’s mother or primary carer was also identified. A research station was set up in each camp and all identified children and their carers available for interviews between July–August 2021 were invited to participate in the research. A local partner guiding the authors around the camps encouraged camp inhabitants to participate in an opportunistic manner. Informed consent was obtained orally from all participants. The process of obtaining consent and all assessments were video-recorded. One researcher administered the assessment via the interpreter, and the other researcher filmed the assessment. These roles alternated in each assessment. Participants were given tobacco, cooking salt, and material for clothes for their participation.

### Sociodemographic factors

Sociodemographic data were provided by the mother/carer and included: age; family size; educational level; whether the mother/carer currently leads a predominantly hunter-gathering life; whether their children go to school and if so, to what type of school; which camp the child lives in; child’s physical disability; child’s alcohol use; child’s tobacco use; child’s drug use. We recorded the presence or absence of the participants’ family members in the camp. The presence of the child/young person’s father in camp was recorded if he currently lived in the same camp as the participating mother/carer (even if the father was no longer in a relationship with their mother).

### Population sample

The overall population sample comprised 140 children and young people aged 3–17 years from the Hadza tribe. SDQ data were collected for all 140 children, whereas the DAWBA was administered only to 107 of these children due to time constraints. Sociodemographic data were collected as part of the DAWBA and are presented in Table 2 for the 107 children (with the exception of age which was collected for all 140). We also provide sample characteristics for a subsample of children aged 5–16 years (n = 83/107). A subsample of Hadza children aged 5–16 years (n = 113/140) was also created to facilitate comparison with SDQ data from the Mental Health of Children and Young People 2017 (MHCYP).

The sample ranged in age from 3 to 17 years with a mean age of 8.50 years (standard deviation =  ± 3.80), a median age of 8 years (interquartile range (IQR) 5–11 years) and 53.3% of the sample were female (n = 57). In terms of living status, 70.1% of children reported living with their mother, and 59.8% reported living with their father. 45.8% of the children lived in Camp 1, 23.4% lived in Camp 2, 14.0% lived in Camp 3, and 16.8% lived in Camp 4. 29% of children had received or were receiving education with a median education duration of 4 years (IQR 2–6 years). No children consumed alcohol regularly, but 5.6% reported using tobacco and 3.7% reported using marijuana. Three children (2.8%) had a physical disability and three had psychological problems according to the Development and Well-Being Assessment (DAWBA).

### Mental health of children and young people in England 2017: comparator group

The Mental Health of Children and Young People in England 2017 (MHCYP) survey is an English national cross-sectional survey involving a stratified multistage random probability sample of children and young people, and their parents and teachers (n = 18,029)^17^. The full methodology is detailed elsewhere^21^.

Sampling was taken from the National Health Service Patient Register as a nationally representative sampling frame. Children and young people were eligible if they were aged 2–19, lived in England, and were registered with a general practitioner. Participants were administered a variety of tests including the Strengths and Difficulties Questionnaire (SDQ)^24^, and the Development and Well-Being Assessment (DAWBA)^25^ to establish the prevalence of psychiatric disorders. The SDQ is the most commonly used validated cross-cultural behavioural screening tool for child and adolescent mental health globally. The DAWBA is a package of interviews, questionnaires and rating techniques which has been used in all English nationwide surveys of children and young people to establish diagnoses of psychiatric disorders. Outcomes were reported according to age groups 2–4, 5–10, 11–16, and 17–19. We used the pooled results from the 5–10 and 11–16 groups as our comparator, as they most closely resemble the age group of the Hadza population. As the Hadza population was largely unaffected by the coronavirus pandemic, we compares data from Hadza children and young people with pre-pandemic data from the MHCYP 2017.The MHCYP data for the SDQ and DAWBA outcomes are only available as summary data, and thus inferential statistics were not possible.

### Measures of mental health and paediatric mental disorders

To establish the prevalence of positive and negative behavioural symptoms, the Swahili parent-report version of the SDQ was administered to all mothers/carers of the sample population. To establish the prevalence of paediatric mental disorders according to the International Classification of Diseases (ICD-10)^26^, the Swahili language parent version of the DAWBA was administered to parents/carers of the sample population. The children themselves were not interviewed. All SDQs and DAWBAs were conducted by two authors (DO and EW) with a local interpreter fluent in both Swahili and Hadzane.

### Statistical analysis

Sociodemographic factors and psychiatric diagnoses were summarised as means and standard deviations, medians and interquartile ranges (IQR), and frequencies. SDQ scores were summarised as means and confidence intervals to allow comparison with SDQ data from the MHCYP 2017. Descriptive results are provided for both the overall Hadza sample (aged 3–17 years) and Hadza children aged 5–16 years to facilitate comparison with MHCYP 2017 data. Cross-sectional associations between sociodemographic factors (age, sex, camp, and which parent they live with) and total and individual domain SDQ scores in the overall sample (aged 3–17 years) were explored with the use of linear regression models.

Multiple imputation using chained equations was performed to deal with missing data on the SDQ. Multiple imputation was based on SDQ items for which there were no missing values^27^.

All statistical analysis were performed using STATA 17.0 (Stata Corp LLP, College Station, TX).

### Ethical approval

King’s College London’s Research Ethics Committee approval was obtained. REC reference number HR/DP-20/21-23691.

### Consent to participate

This study complies with all relevant ethical regulations. The study protocol was approved by an ethics board. We obtained informed consent from all participants, including informed consent from a parent/guardian for study participation by minors.

## Data Availability

The data that support the findings of this study are available from the corresponding author, DO, upon reasonable request.
