# Author Correction: The Prevalence of Behavioural Symptoms and Psychiatric Disorders in Hadza Children

**DOI:** 10.1038/s41598-026-50988-z

**Published:** 2026-05-06

**Authors:** Dennis Ougrin, Emma Woodhouse, Gavin Tucker, Amy Ronaldson, Ioannis Bakolis

**Affiliations:** 1 Youth Resilience Unit, Centre for Psychiatry and Mental Health, Wolfson Institute of Population Health, WHO Collaborating Centre for Mental Health Services Development, Queen Mary University of London, London, UK; 2 Compass Psychology Services, Bromley, Kent, UK; 3 Department of Forensic and Neurodevelopmental Sciences, Institute of Psychiatry, Psychology and Neuroscience, King’s College London, London, UK; 4 South London and Maudsley NHS Foundation Trust, London, UK; 5 Health Service and Population Research Department, Institute of Psychiatry, Psychology and Neuroscience, King’s College London, London, UK; 6 Department of Biostatistics and Health Informatics, Institute of Psychiatry, Psychology and Neuroscience, King’s College London, London, UK

Correction to: *Scientific Reports* 10.1038/s41598-023-48114-4, published online 12 December 2023

The original version of this Article contained an error where identifying participant information was inadvertently published.

As a result, to protect their privacy, the Hadza camps are now referred to numerically as ‘Camp 1’, ‘Camp 2’, ‘Camp 3’ and Camp 4’ in text throughout, along with Table 2 and Table 3. A justification for this has been included in the Study Design section, replacing a sentence that originally named the camps studied.

The original Article has been corrected.

